# Emerging Roles of Nucleoside Transporters

**DOI:** 10.3389/fphar.2018.00606

**Published:** 2018-06-06

**Authors:** Marçal Pastor-Anglada, Sandra Pérez-Torras

**Affiliations:** ^1^Biochemistry and Molecular Pharmacology Section, Department of Biochemistry and Molecular Biomedicine, Institute of Biomedicine (IBUB), University of Barcelona, Barcelona, Spain; ^2^Oncology Program, National Biomedical Research Institute on Liver and Gastrointestinal Diseases (CIBER EHD), Instituto de Salud Carlos III, Barcelona, Spain; ^3^Genetics, Molecular Biology and Gene Therapy Program, Institut de Recerca Sant Joan de Déu, Barcelona, Spain

**Keywords:** nucleoside transporter, hCNT, hENT, transceptor, interactome, nucleoside homeostasis

## Abstract

Since human Nucleoside Transporters (hNTs) were identified by their activity as transport systems, extensive work has been done to fully characterize them at the molecular and physiological level. Many efforts have been addressed to the identification of their selectivity for natural substrates and nucleoside analogs used to treat several diseases. hNTs belong to two different gene families, *SLC28* and *SLC29*, encoding human Concentrative Nucleoside Transporters (hCNTs) and human Equilibrative Nucleoside Transporters (hENTs), respectively. hCNTs and hENTs are integral membrane proteins, albeit structurally unrelated. Both families share common features as substrate selectivity and often tissue localization. This apparent biological redundancy may anticipate some different roles for hCNTs and hENTs in cell physiology. Thus, hENTs may have a major role in maintaining nucleoside homeostasis, whereas hCNTs could contribute to nucleoside sensing and signal transduction. In this sense, the ascription of hCNT1 to a transceptor reinforces this hypothesis. Moreover, some evidences could suggest a putative role of hCNT2 and hCNT3 as transceptors. The interacting proteins identified for hCNT2 suggest a link to energy metabolism. Moreover, the ability of hCNT2 and hCNT3 to transport adenosine links both proteins to purinergic signaling. On the other hand, the broad selectivity transporters hENTs have a crucial role in salvage pathways and purinergic signaling by means of nucleoside pools regulation. In particular, the two new hENT2 isoforms recently described together with hENT2 seem to be key elements controlling nucleoside and nucleotide pools for DNA synthesis. This review focuses on all these NTs functions beyond their mere translocation ability.

## Introduction

The first human Nucleoside Transporter (hNT) protein was identified and cloned more than 20 years ago. In a few more years, the whole panel of hNTs was fully unveiled (Griffiths et al., 1997a,b; Ritzel et al., 1997, 1998, 2001; Acimovic and Coe, 2002; Baldwin et al., 2004). Since then, the study of these proteins has mostly focused on the identification of their substrate selectivity and specificity, their structural determinants and their tissue and subcellular localization.

hNTs are encoded by genes belonging to two different SoLute Carrier (SLC) families, *SLC28* and *SLC29*. *SLC28* include the three members of the human Concentrative Nucleoside Transporter (hCNT1, 2 and 3) family, whereas *SLC29* comprises the four members of the human Equilibrative Nucleoside Transporter (hENT1, 2, 3, and 4) family. NTs are integral membrane proteins, albeit the CNTs and ENTs are structurally unrelated. NTs, or at least some of them, are physiologically relevant mainly due to their likely role in the salvage pathway of natural nucleosides. Moreover, their ability to transport nucleoside analogs currently used as drugs to treat several diseases has also stimulated research on hNTs in the pharmacological field.

In the last few years, several studies on NT biology have contributed to change the focus on these proteins, shifting from the analysis of their role as mere substrate translocators to more diverse functions in cell physiology. The possibility of hCNT showing transceptor functions will be discussed, along with other roles particular NT subtypes can play in cell biology. Overall they support the view that new roles for nucleoside transporters are emerging.

## NTs as Substrate Translocators

### Concentrative Nucleoside Transporters

**FIGURE 1:**
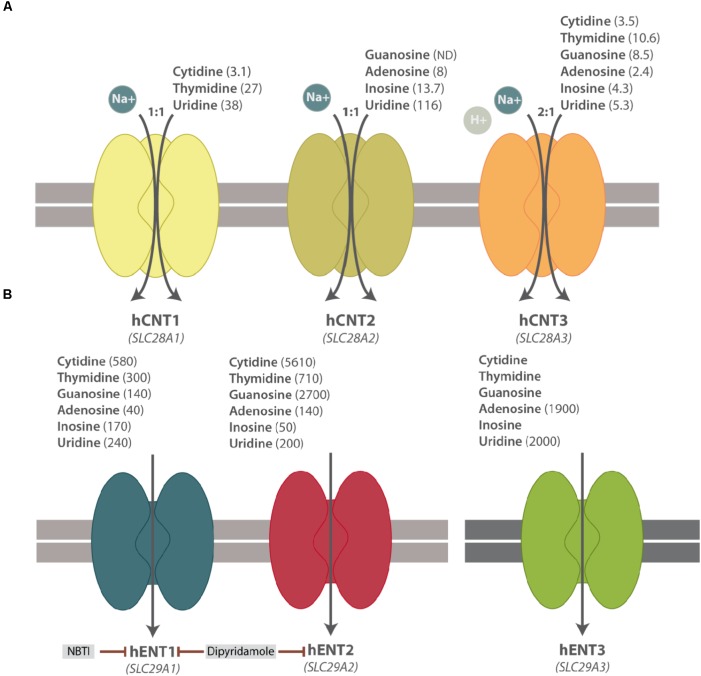
Simplified scheme of hNT protein functions. **(A)** hCNT substrate selectivity and Na^+^-nucleoside coupling ratio. **(B)** hENT substrate selectivity and their pharmacological inhibitors. Apparent affinity constants (μM) of NT substrates are shown in parenthesis. ND means Not Determined.

CNTs mediate the unidirectional uptake of nucleosides coupled to the influx of sodium ions, albeit CNT3 can also accept protons. The sodium/nucleoside coupling ratio is 1:1 for CNT1 and CNT2 and 2:1 for CNT3. The three members also differ among them in their substrate selectivity, except for uridine, which can be transported by all subtypes. CNT1 transports pyrimidines and CNT2 carries purines, whereas CNT3 mediates the uptake of both purines and pyrimidines (**Figure 1A**). The basic biochemical properties (i.e, substrate selectivity and specificity) of NTs have been recently reviewed and summarized elsewhere (Young et al., 2013; Young, 2016; Pastor-Anglada et al., 2018).

hCNTs were initially thought to be restrictively expressed in polarized epithelia, although their expression appears considerably broader than expected. These proteins present a preferential localization at the apical side of polarized epithelia in contrast to ENT proteins, thereby favoring nucleoside transepithelial flux (Mangravite et al., 2001, 2003; Lai et al., 2002; Errasti-Murugarren et al., 2007).

### Equilibrative Nucleoside Transporters

ENTs mediate the facilitative transport of nucleosides and some of them also transport nucleobases, albeit with different specificity. Except for ENT4, ENT proteins have a broad permeant selectivity and are able to transport both purines and pyrimidines (**Figure 1B**) (Young et al., 2013; Pastor-Anglada et al., 2018). These three transporters differ among them by nucleoside specificity although, in general, ENTs present lower affinity for their substrates than the CNT family members. ENT1-3 are widely distributed in different cell types and tissues, even though ENT3 is located at intracellular membranes, mostly in lysosomes but also in mitochondria (Baldwin et al., 2005; Govindarajan et al., 2009). ENT4 is evolutionarily divergent from ENT1-3 and it cannot be considered a conventional nucleoside transporter. In fact, it was originally described as a polyspecific organic cation transporter (Engel et al., 2004), even though it transports adenosine at low extracellular pH (Barnes et al., 2006). The possibility of intracellular pH regulating adenosine transport via hENT1 and hENT2 proteins has been recently reported in HUVECs (Celis et al., 2017).

## NTs Beyond Transport

Both nucleoside transporter families share common features and are often expressed in the same cell types and tissues. It is worth mentioning that ENTs are bidirectional transporters whereas CNTs are not. Moreover, their different substrate specificity and mode of action might support different and probably complementary roles in modulating nucleoside bioavailability. Nevertheless, it also appears that there might be some apparent biological redundancy. This could be associated with additional specific functions and/or diverse regulatory roles for the different NT protein subtypes. In this sense, it can be hypothesized that ENTs might be related to metabolic homeostasis whereas CNTs may be associated with more specialized functions, such as nucleoside sensing and signal transduction.

### The hCNT1 Transceptor

The transceptor concept was presented for the first time by Johan Thevelein back in 1999 in the course of the 14th Small Meeting of Yeast Transport and Energetics (SMYTE) held in Cordoba (Spain) (Diallinas, 2017), although findings supporting this concept were published later (Donaton et al., 2003). Thevelein and colleagues are also reviewing the state of the art of this topic in this issue. The studies of the general amino acid permease Gap1 of *Saccharomyces cerevisiae* evidenced that Gap1 combined transporter and receptor functions (Donaton et al., 2003; Rubio-Texeira et al., 2010). The coexistence of these two functions within the same protein led to coin the word “transceptor” (from **trans**porter and re**ceptor** contraction). Since then, several high affinity solute transporter proteins have been demonstrated to fit under the transceptor concept. In this context, considering the overlapping selectivity and the frequent redundant localization of Equilibrative and Concentrative Nucleoside Transporters, the high affinity CNTs seemed good candidates to be classified as transceptors.

**FIGURE 2:**
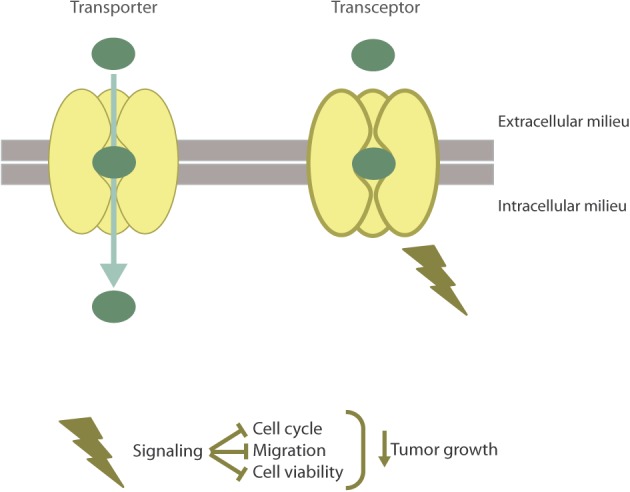
The hCNT1 transceptor. hCNT1, as a result of substrate binding is able to alternate from outward- to inward-facing conformations to allow the translocation of pyrimidine nucleosides into the cell. Aside from the transporter function of hCNT1, this protein is able to act as a nutrient transceptor with no need to translocate. As a transceptor, hCNT1 has been involved in the regulation of cell cycle, migration, cell death, and tumor growth.

Recently, hCNT1 has been the first NT described as a transceptor by our research group (**Figure 2**) (Pérez-Torras et al., 2013). Considering NTs have a major role in determining the bioavailability of several anticancer drugs, various studies have been performed to analyze their expression in cancer. In particular, it has been shown that hCNT1 expression is highly reduced or even lost in different types of tumors (Farré et al., 2004; Gloeckner-Hofmann et al., 2006; Lane et al., 2010; Bhutia et al., 2011; Urtasun et al., 2017). Accordingly, hCNT1 biological function (hCNT1-related transport activity) is almost undetectable or null and its expression at the protein and mRNA level is often lost or dramatically reduced in cancer derived cell lines. Actually, CNT1 is a highly regulated protein (Valdés et al., 2002; Fernández-Veledo et al., 2004; Podgorska et al., 2007; Klein et al., 2009) and its expression has been associated with differentiation (Soler et al., 2001; Soler et al., 2003). Interestingly, restitution of hCNT1 in cancer cell lines provoked unexpected effects that could not be explained by the mere increase in the uptake of pyrimidine nucleosides. Although purine/pyrimidine unbalance can by itself induce a variety of genotoxic effects (Mathews, 2015) the biological impact of hCNT1 restitution, as discussed below, was mimicked by the polymorphic variant hCNT1S546P which is unable to translocate its substrates but appears to be properly folded and inserted into the plasma membrane (Cano-Soldado et al., 2012; Pérez-Torras et al., 2013). hCNT1 restoration in pancreatic cancer derived cell lines significantly altered the phosphorylation status of signal-transducing kinases related to different pathways, reduced cell migration, cell cycle progression and induced non-apoptotic cell death. Moreover, restoration of hCNT1 significantly reduced tumor growth (Pérez-Torras et al., 2013). All these observations, taken together, were crucial to define hCNT1 as a transceptor. Although the physiological relevance of hCNT1 as a transceptor protein is still being studied in our laboratory, the variety of effects observed after its restitution in a cancer background suggests that different signaling pathways might be implicated in hCNT1-induced biological effects, which we think might be, at least partially, related to its interactome.

### hCNT2 and hCNT3 as Putative Transceptors

The two other members of the *SLC28* gene family, hCNT2 and hCNT3, have not been assigned yet as transceptors, although it can be hypothesized they might be.

The study of the hCNT2 interactome allowed the identification of two proteins related to energy metabolism. Glucose-Regulated Protein 58 (GRP58) and aldolase B were identified as hCNT2 partners using the N-terminus tail of hCNT2 as bait (Huber-Ruano et al., 2010). The biological functions of these partners could anticipate a regulatory model dependent on cell nutrient bioavailability. In this sense, GRP58 expression increased in low glucose conditions, whereas hCNT2 expression and activity decreased. Moreover, glucose and fructose availability increased the glycolytic flux and induced transient interactions between aldolase B and hCNT2, which in turn were associated with increased transport activity by a mechanism compatible with transporter conformational changes (Huber-Ruano et al., 2010). Moreover, purinergic modulation of CNT2 in hepatocytes is dependent upon glucose availability and KATP channel function (Duflot et al., 2004). These observations suggest that hCNT2 function is dependent upon energy metabolism, although the possibility of hCNT2 function regulating aldolase B activity by protein–protein interactions cannot be ruled out. To some extent this would be consistent with a putative transceptor role for hCNT2.

On the other hand, the purine nucleoside adenosine is a multifaceted signaling molecule implicated in a variety of physiological and pathophysiological processes. Both hCNT2 and hCNT3 can also modulate cell biology by means of their ability to regulate extracellular adenosine levels and consequently, purinergic signaling. Although this role mainly relies on their transporter activity, a sort of sensor function cannot be ruled out in order to trigger the activation of specific signaling pathways to regulate cell physiology. Indeed, as mentioned above, CNT2 was shown to be under the Adenosine Receptor 1 (A1R) control in hepatocytes by a mechanism mediated by the activation of ATP-sensitive K(+) channels, being the extent of purinergic modulation affected by glucose availability (Duflot et al., 2004). A similar crosstalk between CNT2 and adenosine receptors was also been reported in neurons. Under several adverse conditions characterized by increased adenosine levels such as sleep deprivation and ischemia induced by an experimental ictus, CNT2 expression was down-regulated in the rat brain (Guillén-Gómez et al., 2004; Medina-Pulido et al., 2013). Moreover, adenosine transported by CNT2 is able to trigger AMPK activation in intestinal epithelial, liver parenchymal, and neuronal cell models (Aymerich et al., 2006; Medina-Pulido et al., 2013). The possibility of a single transporter protein mediating both adenosine removal from the extracellular milieu and AMPK signaling is consistent with the view that CNT2 is playing a regulatory role in cell physiology beyond the mere salvage of extracellular nucleosides.

Aside from CNT2, the other high affinity adenosine transporter, CNT3, also contributes to the crosstalk regulation between adenosine transporters and receptors. hCNT3 shows broad expression in epithelial tissues albeit in some cases presents a differential expression pattern to that reported for hCNT2. hCNT3 is poorly expressed in normal hepatocytes, although it is well represented in biliary epithelia, where it appears to be the major player in extracellular adenosine levels regulation. In cholangiocytes CNT3 is under purinergic regulation via the Adenosine receptor 2A (A2AR), thereby contributing to complete purinergic control of bile flow started by ATP secretion into the bile (Godoy et al., 2014).

### ENT2 in Cell Cycle Progression

The broad selectivity transporters ENTs, contrary to CNTs, are widely distributed in all tissues. This feature could suggest a main role of ENTs in salvage pathways required to maintain the nucleoside and nucleotide pools. Indeed, as mentioned above, it is not unlikely that CNT expression is lost in poorly differentiated but highly proliferative cell types. In this regard, the role of these membrane proteins (ENTs) would better fit with the classical roles assumed to be played by most transporters. This is the provision of substrates for either metabolic or structural needs. Nevertheless, regulation of nucleoside and nucleotide pools may also have regulatory relevance. Nucleos(-t)ides are compartmentalized within cells into cytoplasmic and nuclear pools, such amounts being different and dependent on cell requirements (Khym et al., 1978; Bjursell and Skoog, 1980). Moreover, abnormalities in dNTP pool sizes determine DNA replication fidelity and may contribute to mutagenic processes related to carcinogenesis (reviewed in Mathews, 2015). Interestingly, ENTs appeared later in the evolution, being only restricted to eukaryotes which are defined by the presence of nucleus and organelles surrounded by cell membranes. NTs were initially considered to be predominantly plasma membrane proteins (Endo et al., 2007). However, hENT2 and two novel nuclear smaller hENT2 isoforms generated by differential splicing have recently been identified as key elements controlling the nucleoside and nucleotide pool for effective DNA synthesis and cell cycle progression (Grañé-Boladeras et al., 2016). This new evidence clearly points to a model where hENT2 and their isoforms are responsible for the rapid supply of nucleotides required into the nucleus during DNA replication.

### ENT1 and Adenosine Signaling in Physiological and Pathophysiological Conditions

As mentioned above, adenosine is an important component of the purinergic signaling machinery. Considering that ENTs are present in all tissues, including the Central Nervous System (CNS), it is possible that they are members and potential pharmacological targets of purinergic signaling in physiological conditions. Adenosine signaling has been implicated in the pathophysiology of many diseases and psychiatric disorders. Alterations in the function of ENT subtypes able to transport adenosine (mainly ENT1), have been associated with altered adenosine levels thereby resulting in abnormal signaling likely to contribute to several pathophysiological conditions. Mice lacking ENT1 (Choi et al., 2004) have been a very useful model to study the association of this transporter with adenosine signaling. In this regard, owing to the ability of the other ENTs to transport adenosine, their implication in adenosine physiological functions and their pathophysiological alterations cannot be discarded and deserves further research. In the CNS, diminished ENT1, with regards to addictive disorders has been directly associated with increased vulnerability to both goal-directed behavior and excessive ethanol drinking (Choi et al., 2004; Nam et al., 2011, 2013). Furthermore, impaired ENT1 expression and thus adenosine homeostasis results in a reduction in glial fibrillary acidic protein (GFAP), excitatory amino acid transporter type 2 (EAAT2) and aquaporin type 4 (AQP4) expression, and alters astrocyte function (Wu et al., 2010, 2011; Lee et al., 2013; Hinton et al., 2014a). Additionally, either low levels or pharmacological inhibition of ENT1 contribute to cardioprotection due to elevated adenosine circulating levels (Rose et al., 2010; Ramadan et al., 2014). ENT1 has also been associated to biomineralization disorders, its loss contributing to reduced bone density (Hinton et al., 2014b) and to ectopic mineralization of soft tissues (Warraich et al., 2013).

Regarding CNS, animal models and patient samples have revealed that ENT1 and adenosine constitute biomarkers of the initial stages of neurodegeneration in Huntington disease with ENT1 transcript being significantly upregulated in those patients (Guitart et al., 2016). Thus, ENT1 inhibition could be a potential therapeutic target for treating Huntington disease (Guitart et al., 2016, 2017; Kao et al., 2017).

## NTs Interactome

NTs have been often considered as independent entities at the cell membrane with a unique function as nucleoside gatekeepers. Nevertheless, a growing number of studies are contributing to build up a new perspective of NT biology, which we think will be, at least in part, explained by their interacting proteins and the networks they might belong to.

Regarding hCNTs, as previously discussed, the two hCNT2 interacting proteins GRP58 and aldolase B, suggest a link between this particular transporter subtype and energy metabolism (Huber-Ruano et al., 2010). At this moment, these are the most relevant, *a priori* biologically unrelated interacting proteins, described for CNTs. Nevertheless, the transceptor role of hCNT1 would unveil the occurrence of unexpected interactions likely to explain some of its biological actions beyond its mere role as a nucleoside transporter protein. Some other reported interactors will not help to explain putative transceptor functions but might help to understand very basic biological phenomena responsible for hCNT trafficking and turnover. That would be the case for RS1 and galectin-4. The transporter regulator protein RS1 controls the abundance and activity of the three hCNT members at the plasma membrane, particularly in polarized epithelia. Although there is no biochemical evidence of direct interaction between RS1 and selected transporter subtypes, it is highly probable that common regulatory events implicating the three proteins rely upon protein complexes where RS1 and hCNTs are present. This regulatory function seems to be exclusively related to trafficking events resulting in the down-regulation of these proteins without any evident effect of RS1 at the transcriptional level (Errasti-Murugarren et al., 2012). Interaction of hCNT3 with galectin-4 was described in colonic epithelial cells where this partnership appears to be crucial for the proper insertion and retention of this transporter at the apical membrane, determining also hCNT3 turnover (Fernández-Calotti et al., 2016).

Concerning ENTs, the first set of putative ENT1 interactors was identified using a variety of proteomic approaches and, as can be anticipated for the CNTs, these interacting partners include various types of proteins. Among them, calcium signaling transducer calmodulin (CaM), the interactor already confirmed, binds to the large intracellular loop of ENT1 in a calcium-dependent manner (Bicket et al., 2016). In this case, the interaction appeared to explain, at least in part, the calcium-signaling-dependent regulation of nucleoside flux described for neural cells (Zamzow et al., 2009; Wall and Dale, 2013). For the other ENTs, only the interaction of ENT2 with its novel spliced isoforms described above have been published, albeit some interactors may also be envisaged.

## NTs and Disease

NTs are crucial to ensure nucleoside and nucleotide homeostasis and due to the importance of these molecules in many physiological processes it is easy to envisage that their alteration may be behind some pathological conditions.

Cancer is the paradigm of a pathophysiological condition characterized by abnormal high requirement of nucleotides to support DNA synthesis associated to cell proliferation and this may rely upon nucleoside salvage and, accordingly, transport across the plasma membrane. Thus, changes in NTs profile to fulfill nucleotide demand may be expected. However, the situation seems to be more complex. Indeed, expression of hCNT-type transporters is commonly associated with differentiated cell lines and at least for hCNT1, oncogenesis is often related to down-regulation of its expression (Farré et al., 2004; Zollner et al., 2005; Lane et al., 2010; Bhutia et al., 2011; Martinez-Becerra et al., 2012; Mohelnikova-Duchonova et al., 2013). hCNT1 loss may be relevant considering it is a transceptor with additional functions that clearly point to a role in cell cycle progression, migration, and cell death regulation (Pérez-Torras et al., 2013). In contrast, nucleoside influx in cancer cells seems to rely on ENT-type transporters. High levels of hENT2 expression have been correlated with advanced stages in diverse tumor types including hepatocellular carcinoma, mantle cell lymphoma, and ovarian carcinoma (Hartmann et al., 2008; Chen et al., 2010; Bock et al., 2012). Indeed, the role of hENT2 isoforms in sustaining nucleotide availability to proliferate could explain in part this association (Grañé-Boladeras et al., 2016). hENT1 studies are more controversial due to the fact that the patient cohorts used usually include treated patients, which makes it difficult to discriminate between the impact of the treatment from the role of hENT1 *per se* (Pastor-Anglada and Perez-Torras, 2015).

Nucleosides are semi-essential nutrients that can be absorbed from the diet and in this sense the intestine is the first step in their absorption. Inflammatory bowel diseases, such as Crohn’s, show an altered pattern of several transporters, including NTs (Wojtal et al., 2009; Pérez-Torras et al., 2016). The pyrimidine- and purine-preferring transporters, hCNT1 and hCNT2 are downregulated, whereas hENT1 is upregulated. Moreover, other purinome members are also altered, reinforcing again the crucial role of NTs in physiological networks (Pérez-Torras et al., 2016).

Moreover, some diseases such as diabetes, hypertension, and pregnancy diseases involve altered adenosine transporter function, basically associated with ENT activities (Pardo et al., 2013; Salsoso et al., 2017; Sobrevia and Fredholm, 2017).

## Conclusion

NTs have been extensively studied regarding their role as substrate translocators. Indeed they were initially identified by means of their nucleoside transport activity and classified as “transport systems” (Oliver and Paterson, 1971; Pickard and Paterson, 1972). Here we have briefly reviewed the state of the art on the emerging roles of NTs. Current evidence shows they may be active players regulating cell physiology instead of mere nucleoside translocators. hCNTs may show transceptor properties, already demonstrated for hCNT1 whereas ENTs might regulate nucleoside pools and purinergic signaling.

These new roles and others to be discovered in the near future will contribute to better understand the rationale for the apparent biological redundancy of NT expression in most cells and tissues. In this sense, NTs must be considered as members of cellular networks relevant for various biological functions, a concept which is likely to apply to many other SLC-encoded proteins.

## Author Contributions

SP-T conceived the review and wrote the first draft of the manuscript. MP-A critiqued and revised the manuscript. SP-T and MP-A read the final version of the manuscript and approved it for submission.

## Conflict of Interest Statement

The authors declare that the research was conducted in the absence of any commercial or financial relationships that could be construed as a potential conflict of interest.
